# A 15-min non-competitive homogeneous assay for microcystin and nodularin based on time-resolved Förster resonance energy transfer (TR-FRET)

**DOI:** 10.1007/s00216-021-03375-8

**Published:** 2021-06-03

**Authors:** Sultana Akter, Urpo Lamminmäki

**Affiliations:** Biotechnology, Department of Life Technologies, Faculty of Technology, University of Turku, 20520 Turku, Finland

**Keywords:** Cyanobacteria, Cyclic peptide hepatotoxin, Environmental contaminant, Fluorescence resonance energy transfer, Sandwich immunoassay, Immunocomplex antibodies, Immune complex antibodies, Hapten

## Abstract

Simple and rapid methods are required for screening and analysis of water samples to detect cyanobacterial cyclic peptide hepatotoxins: microcystin/nodularin. Previously, we reported a highly sensitive non-competitive heterogeneous assay for microcystin/nodularin utilizing a generic anti-immunocomplex (anti-IC) single-chain fragment of antibody variable domains (scFv) isolated from a synthetic antibody library together with a generic adda ((2S,3S,4E,6E,8S,9S)-3-amino-9-methoxy-2,6,8-trimethyl-10-phenyldeca-4,6-dienoic acid)-specific monoclonal antibody (Mab) recognizing the common adda part of the microcystin/nodularin. Using the same antibody pair, here we report a homogeneous non-competitive assay for microcystin/nodularin based on TR-FRET (time-resolved Förster resonance energy transfer) measurement. The anti-IC scFv labeled with Alexa Fluor 680 and the Mab labeled with europium enabled the FRET process to occur in the presence of microcystin/nodularin. The TR-FRET signal is proportional to the toxin concentration in the sample. The rapid (15 min) homogeneous assay without requiring any washing step detected all the tested nine toxin variants (microcystin-LR, -dmLR, -RR, -dmRR, -YR, -LY, -LF -LW, and nodularin-R). Very good signal to blank ratio (~13) was achieved using microcystin-LR and the sample detection limit (blank+3SD of blank) for microcystin-LR was ~0.3 μg/L (~0.08 μg/L in 80-μL reaction well). The practical application of the TR-FRET assay was demonstrated with water samples spiked with microcystin-LR as well as with environmental water. The average recoveries of microcystin-LR from spiked water ranged from 65 to 123%. Good correlation (*r*^2^ = 0.73 to 0.99) with other methods (liquid chromatography-mass spectrometry and previously reported heterogeneous assay) was found when environmental samples were analyzed. The developed wash-free assay has the potential to play as a quick screening tool to detect microcystin/nodularin from water below the World Health Organization’s guideline limit (1 μg/L of microcystin-LR).

**Graphical abstract:**

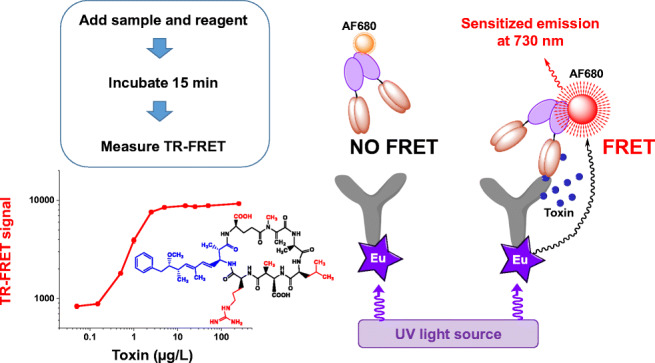

**Supplementary Information:**

The online version contains supplementary material available at 10.1007/s00216-021-03375-8.

## Introduction

Toxic cyanobacterial blooms create local and global problems by contaminating the surface water resources with their potent toxins commonly known as cyanobacterial toxins or cyanotoxins. Microcystins and nodularins are the most commonly reported and troublesome cyanobacterial hepatotoxin having negative effect on animal and human health. Microcystins are also classified as possibly carcinogenic to humans [1]; chronic exposure to trace amounts of toxins has been connected to increased risk of hepatocellular carcinoma [2]. Altogether, microcystins and nodularins have been attributed as causative agents for various animal poisonings and identified as a threat for human health [3–5].

Though microcystins are found in freshwater bodies of all over the world and nodularins in predominantly less salty coastal brackish water (e.g., the Baltic Sea, coastal water of Southern Australia) [6], they share structure similarities. Both are monocyclic peptides; microcystins are composed of seven amino acids while nodularins are composed of five amino acids. An unusual β-amino acid adda ((2S,3S,4E,6E,8S,9S)-3-amino-9-methoxy-2,6,8-trimethyl-10-phenyldeca-4,6-dienoic acid), which is essential for toxicity, is present in both microcystins and nodularins among with few other amino acid similarities. Structural variation occurs in both of them; however, it is much more prevalent in microcystins than in nodularins. Until now, over 250 microcystin congeners are reported in the literature along with about 10 congeners of nodularin [7, 8]. However, in the natural environment, many of these variants are present in minute quantities among the frequently reported microcystin congeners, such as microcystin-LR, -RR, -YR- LA, -WR, and dimethyl microcystin-LR, -RR depending on the geographical distribution [6, 9]. Microcystin-LR is the most commonly reported and widely distributed toxic microcystin variant. For nodularins, nodularin-R is the most dominant variant [10].

In 1998, WHO (World Health Organization) recommended a provisional guideline value of 1 μg/L of microcystin-LR in drinking water [11]. Recently, WHO revised the provisional guideline values of microcystin in drinking water and included recommended guideline values for recreational water. The guideline values for lifetime drinking water, short-term drinking water, and recreational water are 1 μg/L, 12 μg/L, and 24 μg/L of microcystin-LR equivalent [9]. WHO recommends that the public should be informed about cyanobacterial blooms in source waters when the water is used for recreation or for producing drinking water [9]. Simple and efficient methods for sensitive and quick screening within or below the WHO guideline level (1–24 μg/L of microcystin-LR equivalent for drinking water) are particularly in high demand.

Until now, there is no single analysis method sufficient alone for cyanotoxin monitoring. Existing analytical methods such as high-performance liquid chromatography (HPLC) or mass spectrometry (MS) are time consuming, expensive, and require expertise. Some methods (mouse bioassay) are cumbersome and involve animal sacrifice, and many lack specificity and sensitivity (protein phosphatase inhibition assays, PPIAs) [12]. Immunoassays with sufficient sensitivity and specificity are a promising alternative method for microcystin/nodularin detection. Immunoassays are simple and easy to perform, and raw water can be directly applied to the assay without any sample processing. Thus, simple immunoassays are particularly promising tools for fast screening of large number of samples.

However, the available immunoassays (including commercial ones) for microcystin and/or nodularin are generally in the form of competitive format [13] as usual with immunoassays for low molecular weight targets (MW less than 2000 Da). Relying on one antibody recognition site, competitive assays are time consuming (2–3 h), requiring several incubation and washing steps, which must be strictly maintained. Furthermore, the generated signal is inversely proportional to the analyte concentration, which can complicate the interpretation of the result.

We have previously reported the development of sensitive non-competitive immunocomplex principle-based immunoassays for microcystin/nodularin [14, 15]. These assays utilize a unique antibody pair consisting of a monoclonal antibody binding to the adda group common in microcystin and nodularin, and an anti-immunocomplex (anti-IC) single-chain antibody fragment (scFv) recognizing the anti-adda monoclonal antibody (Mab) when bound to basically any microcystin or nodularin (at least those 11 tested so far) [14]. This unique generic anti-IC scFv was isolated from our synthetic antibody library [16, 17] by phage display. Both the previously developed assays [14, 15] are heterogeneous in nature, requiring an intermediate washing step to remove the unbound components before the signal development step.

Homogeneous immunoassays lacking the washing or separation steps are very appealing detection tools as they provide significant advantages regarding simplicity, rapidity, and less instrumentation requirement. As previously demonstrated by Pulli et al. [18] and Arola et al. [19], the fact that the two antibodies involved in the immunocomplex-based recognition of a low molecular weight analyte are inevitably brought into very close interaction provides an excellent basis for the utilization of the fluorescence/Förster resonance energy transfer (FRET) process for the signal generation in a homogeneous assay [20, 21]. In the FRET process, energy is transferred from a light-excited donor fluorophore through a dipole-dipole coupling interaction to an acceptor fluorophore, which then releases the energy as light at higher wavelength [20, 22, 23]. As the efficiency of the FRET process is highly dependent on the distance, being inversely proportional to the sixth power of it, the efficient energy transfer can typically only occur when the fluorophores are situated not more than 1–10 nm apart. By having the two antibodies needed for the immunocomplex formation labeled with the donor and acceptor fluorophore, respectively, FRET signal is likely obtained upon the recognition of the analyte by the antibodies. In time-resolved fluorescence/Förster resonance energy transfer (TR-FRET) [24], a lanthanide ion–containing chelate is used as the donor compound. Due to the long fluorescence lifetime of such compounds, even >1000 μs [25], the FRET signal can be measured within an appropriate time window after the excitation, which helps to avoid interference due to the short lifetime auto-fluorescence or cross-talk between the fluorophores. This can result in significantly higher signal-to-background ratios and eventually improved assay sensitivity as compared to the standard FRET process.

Here, we describe a TR-FRET-based homogeneous non-competitive sandwich-type immunoassay for the detection of microcystin/nodularin using the aforementioned immunocomplex forming antibody pair: anti-adda Mab and anti-IC scFv (as fused to bacterial alkaline phosphatase). The assay enables us to have a sensitive and quantitative detection of microcystin/nodularin in a simple mix-and-measure approach, in a short time (<15 min). The assay is applicable for quantitative analysis of microcystin-LR with a sample detection limit of ~0.3 μg/L, satisfying the WHO guideline limit (1 μg/L), thus providing a powerful tool for rapid and sensitive screening for cyanobacterial cyclic peptide hepatotoxins.

## Materials and methods

### Common materials and reagents

Colorless assay buffer solution used in the TR-FRET assay, colored assay buffer used in assays other than the FRET assay, 96-well streptavidin-coated plates, wash concentrate for washing the 96-well plate, and enhancement solution to dissociate the lanthanide (europium) ion were from Kaivogen (Turku, Finland). MaxiSorp 96-well microtiter plates used in the FRET immunoassay were from Nunc A/S, Thermo Fisher Scientific (Roskilde, Denmark). Monoclonal antibody AD4G2 (adda specific) which binds microcystin or nodularin through the adda residue was purchased from Enzo Life Sciences, Inc. (USA). Bacterial anti-alkaline phosphatase polyclonal antibody (bAP Pab) purchased from LifeSpan Biosciences, Inc. (USA), was purified and labeled with europium (Eu-bAP Pab) to be used as a tracer in the immunocomplex (IC) assay based on time-resolved fluorescence (TRF) (referred here IC-TRF assay) according to an earlier report [14]. The near-infrared fluorescent label Alexa Fluor® 680 succinimidyl ester (AF680) were purchased from Molecular Probes, Invitrogen (Thermo Fisher Scientific Inc.). The immunoassays were carried out in room temperature (RT) of around 23 °C.

### Instruments

Delfia Plateshake for the shakings of the 96-well plates, plate washer, and enhancement solution dispenser were from Wallac, PerkinElmer Life and Analytical Sciences (Finland). Protein concentration was measured by a NanoDrop ND1000 spectrophotometer (Thermo Fisher Scientific Inc.).

### Plate readers

The luminescent signal of Eu(III) using time-resolved mode was measured by the multilabel counter Victor™ 1420 (PerkinElmer Life Sciences, Finland) by applying default factory settings (excitation 340 nm, emission 615 nm, delay 400 μs, gate time 400 μs). For TR-FRET measurement, the instrument was installed with a red-sensitive photomultiplier tube (R4632, Hamamatsu Photonics, Hamamatsu, Japan) and 730-nm bandpass emission filter of 10-nm bandwidth and 70% transmission maximum (Nabburg, Interferenzoptik Elektronik GmbH, Germany). TR-FRET was measured at 730 nm using a 340-nm excitation wavelength. The delay time of 75 μs and the measurement window of 50 μs were used according to the previous reports [26–28].

### The donor (Eu-anti-adda Mab)

**Fig. 1:**
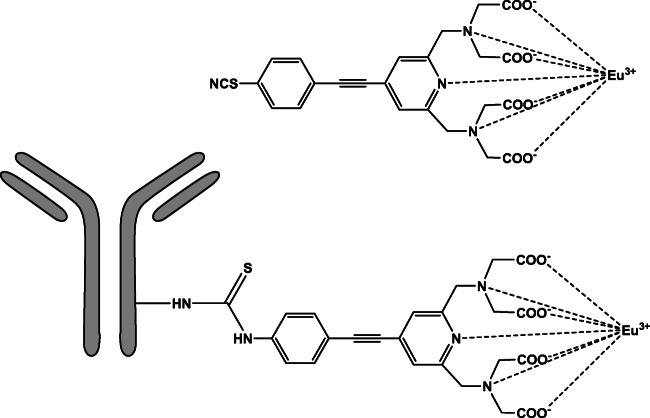
An intrinsically luminescent seven-dentate europium (7d-EuIII) chelate, MW: 674.46 g/mol [29] was used to label the anti-adda Mab to be used as a donor (Eu-anti-adda Mab) in the TR-FRET assay

An intrinsically luminescent seven-dentate europium (7d-EuIII) chelate, [2,2′,2″,2″′-[[4-[(4-isothiocyanatophenyl)ethynyl]pyridine-2,6-diyl]bis(methylenenitrilo)] tetrakis(acetato)-europium(III)], MW 674.46 g/mol [29], was labeled with the anti-adda Mab to be used as a donor in the FRET assay (Fig. 1). The 7d-EuIII chelate was synthesized earlier in the University of Turku (28) according to a previously described method [29]. The anti-adda Mab (~700 μg = ~ 4.38 nmol) and a 100-fold molar excess of 7d-EuIII chelate were dissolved into a total volume of 438 μL of 50 mM carbonate buffer, pH 9.8. The labeling reaction was incubated overnight at +4 °C protected from light. The labeled antibody (Eu-anti-adda Mab) was purified with gel filtration using a Superdex 200 column and eluted in TSA buffer (50 mM Tris-HCl, 150 mM NaCl, and 0.5 g/L NaN_3_), pH 7.75. The chelate concentration in the labeled Eu-anti-adda Mab was measured by comparing the fluorescence of the purified product against a known Eu(III) standard. The labeled antibody concentration was estimated by assuming that 90% of the initial unlabeled antibody was recovered in the collected labeled antibody pool (monitored by absorbance at 280 nm). In the purified product, DTPA (diethylenetriamine pentaacetate)-treated BSA (bovine serum albumin) was added to a final concentration of 0.1% and filtered through 0.22 μm and stored at +4 °C.

### The acceptor

#### Anti-IC scFv-AP

A unique anti-IC scFv SA51D1 binder [14] was previously isolated from synthetic ScFvP antibody library [16, 17] by applying phage display technology using immunocomplex (anti-adda Mab bound to microcystin-LR) in the selection process. The selection process and screening of this binder have been described earlier [14]. The scFv as fusion with bacterial alkaline phosphatase (scFv-AP) was expressed in *Escherichia coli* strain RV308 in laboratory scale (5 L) fermentation at 26 °C. The scFv-AP was purified through ammonium sulfate precipitation, affinity chromatography (HisTrap Fast Flow Ni-NTA column, GE, USA), and size exclusion chromatography (Superdex 200 column, GE, USA) and eluted in TSA buffer, pH 7.5.

#### Conjugations of anti-IC scFv-AP with acceptor fluorophore

The anti-IC scFv-AP was labeled with the near-infrared fluorescent label Alexa Fluor 680 (AF680) to be used as an acceptor fluorophore in the FRET assay. The buffer of the purified scFv-AP was changed into PBS buffer pH 7.4 and then conjugated with AF680 using a reaction between the succinimidyl ester on the AF680 and the primary amino group on the scFv-AP. Aliquots of each 350 μg scFv-AP were mixed with either 5, 8, 10, or 15-fold (batch 1, 2, 3, and 4, respectively) molar excess of AF680 (dissolved in N,N-dimethylformamide from Sigma-Aldrich) in 50 mM carbonate buffer, pH 9.3 in 500-μL volume for 1 h at room temperature. The labeled products were purified by double gel filtration using NAP5 and NAP10 columns (GE Healthcare, UK) and eluted in TSA buffer, pH 7.5. According to the manufacturer’s instruction, labeled protein concentration (M) was measured as [(A_280_ – A_679_ × 0.05) × dilution factor]/203,000, where the molar extinction coefficient of IgG is approximately 203,000 cm^−1^ M^−1^ and correction factor for absorption of the AF680 dye at 280 nm is 0.05. The labeling degrees [(A_679_ × dilution factor) / (184,000 × protein concentration (M)) where the approximate molar extinction coefficient of the AF680 dye at 679 nm is 184,000 cm^−1^ M^−1^] of the purified products were measured by absorbance together with appropriate wavelength and molar absorptivity of the AF680 (provided by the manufacturer). The absorption maximum for unconjugated AF680 dye (MW ~1150) is 679 nm and the emission maximum is 702 nm. For resulting AF680 conjugates, the theoretical absorption maximum is 684 nm and the emission maximum is 707 nm.

#### BSA coating of microtiter wells

To prevent non-specific binding, low-fluorescence yellow 96-well MaxiSorp microtitration plates (Nunc, Roskilde, Denmark) were coated with BSA with saturation solution containing 0.1% BSA (Bioreba, Switzerland) in the presence of 0.1% (w/v) Germall II (ISP, Wayne, NJ) and 3% (w/v) trehalose (Sigma-Aldrich, St. Louis, MO) in 0.05 M Tris-HCl, pH 7.2. Briefly, 250 μL/well of saturation solution was added and incubated for 1 h at room temperature with slow shaking followed by aspiration of liquid. Plates were dried for 2 h and stored at +4 °C in a sealed bag until used in the FRET immunoassay.

#### Homogeneous FRET assay and optimization of assay parameters

The homogeneous assays were performed using 7d-EuIII chelate–labeled anti-adda Mab (Eu-anti-adda Mab) as a donor and fluorescent acceptor dye AF680 conjugated to anti-IC scFv-AP (AF680-scFv-AP) as an acceptor. In BSA-coated microtiter wells, toxin standard (0–100 μg/L of microcystin/nodularin) or sample was added followed by addition of reagent mixture (comprising Eu-anti-adda Mab and AF680-scFv-AP). Wells were then incubated (in room temperature with low shaking), and upon excitation at 340 nm, the sensitized emissions from AF680 generated by FRET were measured at 730 nm by a Victor instrument.

Combination of different amounts of Eu-anti-adda Mab (5–200 ng/well) and AF680-scFv-AP (10–200 ng/well) in a reagent mixture, effect of incubation time (2–60 min), and effect of reaction volume (60–100 μL) were tested on assay performance using microcystin-LR as standard. In addition, combination of different delay times (50–125 μs) and measurement windows (25–50 μs) were explored as measurement parameters.

Finally, in the optimized assay, 20 μL of sample/standard was mixed with 60 μL of reagent mixture (15 ng of Eu-anti-adda Mab and 120 ng of AF680-scFv-AP per well) and incubated for 15 min, and FRET measurement was carried out using 50 μs of measuring time with 75 μs of delay time.

The detection limit (the smallest detectable toxin concentration in the sample) was calculated from the standard curve based on the average response of blank + 3 times standard deviation of the blank. Concentrations of unknown samples were determined from the standard curve with the help of Origin software (OriginLab Corporation, Wellesley Hills, USA).

#### Performance of different AF680-labeled scFv-AP

Four batches (batch 1, 2, 3, 4) of AF680-labeled scFv-AP (AF680-scFv-AP) were prepared using different excess (5x, 8x, 10x, 15x respectively) of AF680. All four batches of AF680-scFv-AP were compared for their performance in preliminary TR-FRET assay using microcystin-LR as standard. In BSA pre-coated plate, 80 μL of reagent mixture containing Eu-anti-adda Mab (0.25 μg/mL) and each batch of AF680-scFv-AP (2.5 μg/mL) were added in the presence of 20 μL of microcystin-LR. In the final 100-μL reaction volume, concentration range of microcystin-LR was 0–40 μg/L. TR-FRET measurement was performed after 5, 10, 15, 20, 25, and 30 min with the delay time of 75 μs and the measurement window of 50 μs.

#### Effect of incubation time on assay performance

The effect of incubation time on the performance of TR-FRET assay was observed using microcystin-LR as standard in duplicate wells. The assay was performed in 80-μL total reaction volume where 20 μL of microcystin-LR standard and 60 μL of reagent mixture were added. Microcystin-LR concentration in the final 80-μL reaction well ranged from 0, 0.05, to 250 μg/L. Signal was measured at different incubation time points: 2 min to 60 min.

#### Standard curves of different microcystin/nodularin variants

A total of nine different purified microcystin variants and nodularin (microcystin-LR, 3-demethylmicrocystin-LR, microcystin-RR, 3-demethylmicrocystin-RR, microcystin-YR, microcystin-LY, microcystin-LF, microcystin-LW, nodularin-R) were (final concentration range in the 80-μL reaction well: 0–250 μg/L) analyzed to determine the specificity profile of the homogeneous TR-FRET assay. Toxins in the form of a lyophilized dried powder were obtained from Dr. Jussi Meriluoto’s Lab (Åbo Akademi University, Turku, Finland) which were initially dissolved in 50% methanol (100–250 μM original stock). The successive working stocks and standard preparation were performed with reagent water. Toxin stocks were kept at −20 °C or 4 °C.

#### Heterogeneous IC-TRF assay

To compare the performance of the homogeneous assay to a similar heterogeneous assay, the environmental samples were also measured using the previously reported IC-TRF assay [14]. The assay concept was previously described [14] and was performed here with the following modification. In streptavidin-coated microtiter wells (Kaivogen, Turku, Finland), microcystin-LR standard (0–20 μg/L) and water samples were added as 20 μL/well in the presence of 60-μL reagent mixture (1 μg/mL of biotinylated anti-adda Mab, 1 μg/mL of scFv-AP, and 0.5 μg/mL of Eu-bAP Pab). The wells were incubated for 1 h at room temperature with slow shaking and washed four times. Then, 200 μL/well of enhancement solution (Kaivogen, Turku, Finland) was added, and after 5–10-min incubation at room temperature with shaking, TRF of Eu signal was measured with a Victor 1420 multilabel counter (Wallac, PerkinElmer Life and Analytical Sciences) using standard europium protocol where excitation wavelength was 340 nm and measurement wavelength was 615 nm.

#### Detection of microcystin-LR from spiked water samples

Reagent water (Millipore) from the laboratory and two raw water samples from different Finnish lakes (Paalijärvi, Riihimäki, Finland 5.8.2009 and Tuusulanjärvi, Tuusula, Finland 24.6.2009) were selected for spiking experiment. The collected environmental samples were stored at −20 °C until use. Before spiking, all samples were tested for any possible presence of toxin by the heterogeneous IC-TRF assay [14]. Each sample was spiked with microcystin-LR (concentration range: 0, 0.2, 0.5, 1, 5, 10 μg/L). Toxin concentrations of the spiked and the corresponding unspiked water samples were measured by the TR-FRET assay using duplicate wells. The recovery was calculated as follows: %*R* = (spiked sample result by TR-FRET − unspiked sample result by IC-TRF assay)*100/known spike added concentration.

#### Analysis of environmental water samples

In total, 18 environmental samples collected during 2009 from Finland and Estonia [30] were analyzed by the developed TR-FRET-based homogeneous sandwich assay. For each sample, there were two parallel sets. One set comprising raw water as such (containing extracellular and any cell-bound toxin) was measured with the current TR-FRET assay as well as with the previously reported heterogeneous IC-TRF-based immunoassay [14]. Stored (−20 °C) raw samples were thawed-freeze-thawed to release the cell-bound toxin and used in the immunoassay without any concentration or further processing steps.

Another set of parallel water samples was previously filtered, toxins were extracted from the collected cells, and aliquots were made. Commercial ELISA and LC-MS were previously performed to determine the extracted intracellular microcystin/nodularin concentration and reported earlier [30]. From this same extracted sample set, one subset of aliquots was stored at −20 °C as dried form (the liquid was evaporated) until analysis by the current TR-FRET assay as well as by the heterogeneous IC-TRF immunoassay. Before analysis, the samples were reconstituted in reagent water accordingly based on known results [30].

## Results

### The developed TR-FRET assay concept

**Fig. 2:**
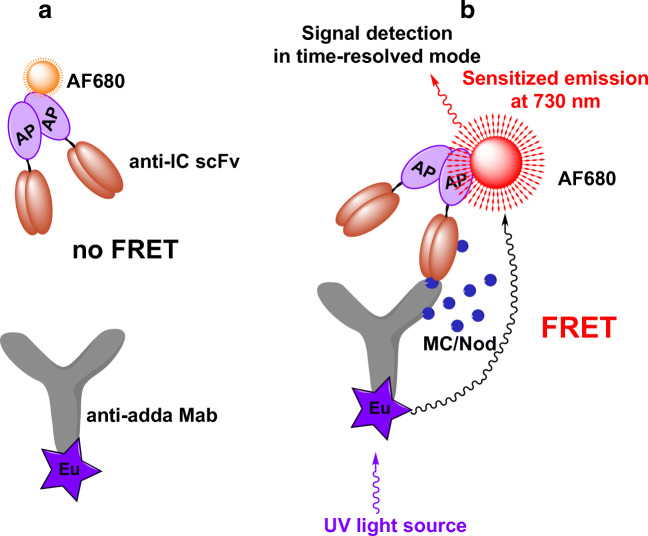
The homogeneous non-competitive TR-FRET immunoassay concept for cyanobacterial cyclic peptide hepatotoxins microcystin (MC) and nodularin (Nod). Eu-chelate-labeled adda-specific monoclonal antibody and AF680-labeled anti-IC scFv-AP are added together with water sample. In the absence of toxin in water sample (**a**), the antibodies are free in the solution and FRET is not detected. In the presence of toxin (**b**), the anti-IC scFv binds specifically to the immunocomplex of anti-adda Mab and MC/Nod, bringing the labels in close proximity. Excitation of the Eu-chelate with UV light results in FRET between the labels, and sensitized emission of fluorescence signal can be detected at 730 nm in time-resolved mode

To develop the TR-FRET-based homogeneous assay for microcystin/nodularin, the broad specific anti-IC scFv as fused to bacterial alkaline phosphatase (AP) was labeled with AF680 fluorophore (AF680-scFv-AP) and the adda-specific monoclonal antibody with the Eu^3+^-chelate (Eu-anti-adda Mab). Figure 2 shows the principle of the developed assay.

### Performance of different AF680-labeled scFv on MC LR standard curve

**Fig. 3:**
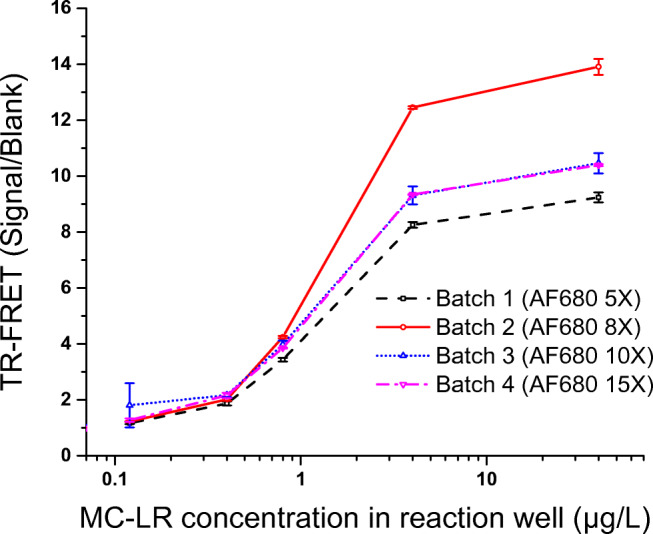
TR-FRET signal to blank ratio of the homogeneous assay using different batches of AF680-labeled scFv-AP in the presence of microcystin-LR in total 100-μL reaction well. TR-FRET measurement of the sensitized emission of AF680 at 730 nm was performed after 15-min incubation. Error bar indicates the standard deviation (SD) of the value

In order to find a suitable labeling degree, four different batches of AF680-scFv-AP were prepared and compared for their performance in the TR-FRET assay using microcystin-LR as analyte (0–40 μg/L in final 100-μL reaction well). A graph representing 15-min incubation data is shown in Fig. 3. All the batches were found to be active and performed well in the homogeneous assay. Batch 2 labeled with 8x molar excess of AF680 performed best in terms of low background and highest signal/blank (S/B) ratio. The top three best performing label batches (labeling with 8x, 10x, and 15x molar excess of AF680) were utilized in the subsequent assays. When we compared the data in terms of signal/blank ratio, batch 2 likewise provided the highest S/B ratio of 13.9 at 40 μg/L of microcystin-LR concentration. At the same toxin concentration, the rest of the label batches contributed similar S/B ratio (9.2 to 10.5). At lower concentration levels (0.8 μg/L of microcystin-LR in reaction well), the performance ranking was batch 2 (S/B 4.2) > batch 3 (S/B 4) > batch 4(S/B 3.9) > batch 1(S/B 3.4).

### Optimization of assay components and measurement parameters

In order to optimize the reagent component in TR-FRET assay, varying amounts of Eu-anti-adda Mab (5–200 ng/well) and AF680-scFv-AP (10–200 ng/well) were evaluated in the assay in ~100-μL reaction well (see Supplementary Information (ESM), Fig. S1). Maximum average signals of 75,049 and 14,962 in the presence of 50 μg/L and 1 μg/L of microcystin-LR respectively were achieved using the highest tested Mab and scFv amounts (200 ng + 200 ng). However, in such case, the blank signal (10,607) is also increased reducing the corresponding signal to background ratios (S/B) (7.1 and 1.4 in the presence of 50 μg/L and 1 μg/L of microcystin-LR respectively). Decreasing amounts of Eu-Mab while maintaining the increased amount of AF680-scFv in a reaction well proportionately decreased the background signal. For example, with 20 ng of Eu-Mab + 200 ng of AF680-scFv, the S/B improved (15.2 and 4.8 in the presence of 50 μg/L and 1 μg/L of microcystin-LR respectively) more than twice. Several combinations of Eu-Mab + AF680-scFv provided S/B above 12 (at 50 μg/L of microcystin-LR) such as 10 ng Eu-Mab + 80 to 160 ng AF680-scFv or 20 ng Eu-Mab + 160 to 200 ng AF680-scFv.

**Fig. 4:**
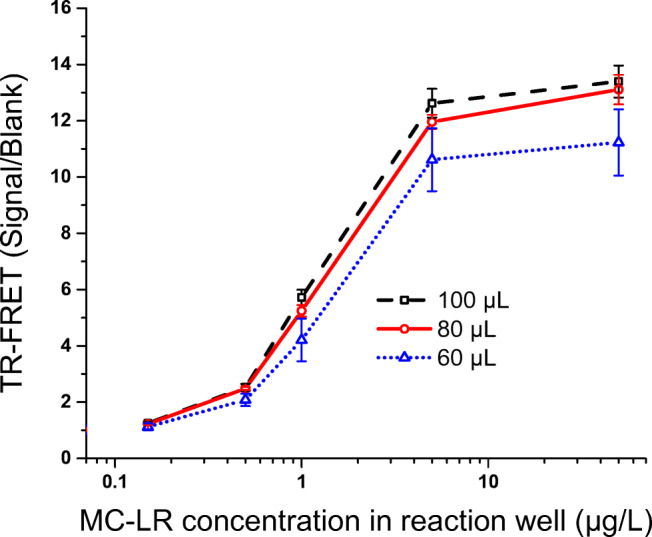
Effect of total reaction volume in the microtiter well in the homogeneous assay using microcystin-LR as standard. Error bar indicates the standard deviation (SD) of the value

Subsequently, the total reaction volume of 60 μL, 80 μL, or 100 μL was explored (Fig. 4) in the homogeneous assay using 15 ng of Eu-Mab and 160 ng of AF680-scFv. TR-FRET signal increased proportionately with the increase of reaction volume. Compared to S/B of 60 μL, the S/B of 80–100 μL was improved. However, the S/B remained more or less similar in case of 80-μL volume or 100-μL volume. Better coefficient of variation % (cv%) of the signal from the duplicate measurements was achieved with 80-μL (cv% range: 0.7 to 3.5%) or 100-μL (cv% range: 1.7 to 3.7%) reaction volume, compared to the 60-μL volume reaction wells (cv% range: 2.5 to 14.9%).

In the Victor fluorometer instrument, for TR-FRET measurement protocol, combination of different counting delay times (50–125 μs), measurement window time (25–50 μs), and flash energy level (EF; 130, 200, and default high 255) were explored to find out the suitable measurement parameters. Among these tested parameters [delay time/measurement window (EF)], measurement at 75/50 (EF high), 50/25 (EF high), and 75/25 (EF 200) seems to deliver best S/B.

Eventually, considering the signal level, S/B level, and acceptable cv%, in addition to minimal reagent consumption, in the subsequent experiments, 80-μL reaction volume was used. In such condition, 20 μL of sample/standard and 60 μL of reagent component comprising ~15 ng/well of Eu-anti-adda Mab and ~120 ng/well of AF680-scFv-AP were used. The measurement protocol included flash energy level of 255, counting delay time of 75 μs, and counting window time of 50 μs.

### Effect of incubation time

**Fig. 5:**
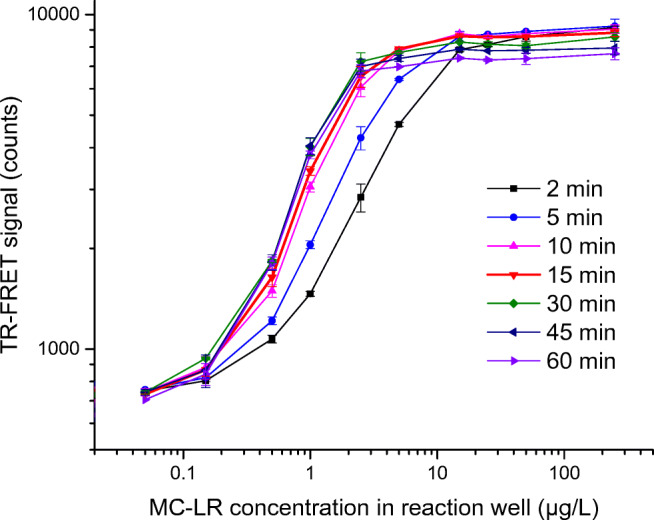
Effect of incubation time on the homogeneous assay performance using microcystin-LR (MC-LR) as standard. In BSA-coated microtiter wells, MC-LR (final concentration in 80-μL reaction well: 0.05 to 250 μg/L, plotted in logarithmic scale in *X* axis) was used to generate TR-FRET signal of the sensitized emission of AF680 at 730 nm (plotted in logarithmic scale in *Y* axis) at different incubation time points (2–60 min). Standard deviations of duplicate measurements are shown as error bar. Error bars are not visible when interfering with symbols

The effect of incubation time on the performance of FRET assay (Fig. 5) was observed using microcystin-LR as standard (toxin concentration in 80-μL reaction well: 0.05 to 250 μg/L). Signal tends to increase up to 10–15-min incubation. Incubating longer than 30 min does not have any beneficiary effect, rather the overall signal tends to drop. Longer incubation, such as 1 h, reduces the total signal level at highest concentration of standard curve. Based on these findings, 15-min incubation time was used for further assays.

### Standard curves of different microcystin/nodularin variants

**Fig. 6:**
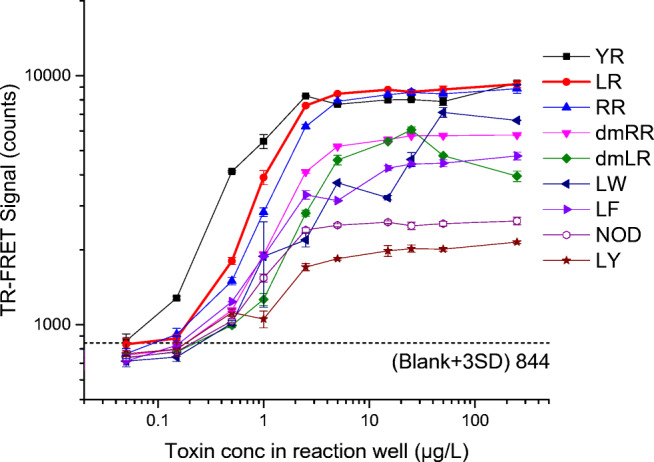
Standard curves of eight different microcystin variants and nodularin-R in the TR-FRET assay. Toxin concentrations in total 80-μL reaction well (0.05 to 250 μg/L) are plotted in logarithmic scale in *X* axis vs the corresponding TR-FRET signal (sensitized emission of AF680 at 730 nm) in logarithmic scale in *Y* axis. Standard deviations of duplicate measurements are shown as error bar. Error bars are not visible when interfering with symbols

Nine different purified toxin analogues [microcystin-LR, 3-demethylmicrocystin-LR, microcystin-RR, 3-demethylmicrocystin-RR, microcystin-YR, microcystin-LY, microcystin-LF, microcystin –LW, and nodularin-R (final concentration range in 80-μL reaction well: 0–250 μg/L)] were analyzed by the homogeneous assay, and the standard curves of the toxin variants are shown in Fig. 6. For the most important variant, microcystin-LR, based on average blank signal + 3 times the standard deviation of 40 blank wells, sample detection limit of ~0.3 μg/L (~0.08 μg/L in reaction well) was achieved. For two other common congeners, microcystin-RR and microcystin-YR, the detection limit is similar or even better than that of microcystin-LR, respectively. For the rest of the microcystin variants and nodularin-R sample, the detection limit is close to or below 1 μg/L. As expected, due to the nature of the immunocomplex assay, no high-dose hook effect was observed, except for 3-demethylmicrocystin-LR (dm-LR), in which case, some decrease in signal occurs at concentration above 25 μg/L.

### Recovery with spiked water sample

Reagent water and two Finnish lake water samples (Paalijärvi and Tuusulanjärvi) were used for spiking with microcystin-LR at concentrations 0 to 10 μg/L. In the non-spiked lake waters, some amount of toxin (extracellular and/or intracellular toxin) was detected (Paalijärvi: 0.09 μg/L and Tuusulanjärvi: 0.17 μg/L) according to IC-TRF assay [14]. The spiked water was used in the TR-FRET immunoassay directly without any concentration or further dilution steps. Table 1 shows the measured toxin concentration and the recovery percentage of the sample by the TR-FRET measurements. While the TR-FRET assay was, as expected, not able to detect the lowest spiked toxin concentration (0.2 μg/L), the recoveries from samples spiked with 0.5 to 10 μg/L toxin were satisfactory and ranged from 65 to 123%. For these samples, the coefficient of variation (cv %) of TR-FRET measurement was also in the acceptable range (2 to 20%). The results clearly demonstrated that the assay is applicable for measuring water sample below and near the WHO guideline value (1 μg/L of microcystin-LR) for drinking water.

**Table 1:** Analysis of toxin-spiked water samples by TR-FRET assay

	MQ			Paalijärvi (5.8.2009)			Tuusulanjärvi (24.6.2009)		
MC-LR added to the sample (μg/L)	Toxin conc by TR-FRET (μg/L)	Recovery (%)	CV (%)	Toxin conc by TR-FRET (μg/L)	Recovery (%)	CV (%)	Toxin conc by TR-FRET (μg/L)	Recovery (%)	CV (%)
0	>dl	-	-	>dl (0.9)a	-	-	>dl (0.17)a	-	-
0.2	>dl	-	-	>dl	-	-	>dl	-	-
0.5	0.6	115	9	0.6	101	15	0.5	65	20
1	1.0	96	9	1.1	99	6	1.2	100	16
5	5.0	100	2	5.3	105	2	5.8	113	2
10	11	110	8	12.1	121	6	12.5	123	14

Coefficient of variations % (cv %) are of two replicate measurements. >dl (below detection limit); a, toxin concentration detected according to IC-TRF assay [14]

### Environmental sample analysis

A total of 18 environmental water samples collected from Åland island of Finland, mainland Finland, and Estonia were analyzed with the TR-FRET and heterogeneous IC-TRF assays to determine the microcystin/nodularin concentration present in the natural raw water (intracellular and extracellular) as well as in the extracted sample (intracellular). The results are shown in the Table 2. Before analysis, each raw water samples were frozen and thawed at least twice to release any possible intracellular toxin in the water. For each sample, a corresponding parallel lyophilized sample set comprising extracted intracellular toxin was available which was reconstituted by water and analyzed also by TR-FRET and IC-TRF assays. For these samples, intracellular toxin concentration results by LC-MS were also available from a previous publication [30]. Amount of toxin in raw water was higher than the extracted intracellular toxin in many samples. This is an expected phenomenon as the raw water contains both the cellular toxin and the already released extracellular toxin. Good correlation was found between TR-FRET and the reference measurement assays. For toxin measurement from raw water, the coefficient of determination (*r*^2^) between TR-FRET and IC-TRF assay was as high as 0.99. For intracellular toxin measurement, *r*^2^ was 0.73 and 0.75 in respect to IC-TRF assay and LC-MS analysis, respectively.

**Table 2:** Microcystin/nodularin amount in the environmental water samples from Finland and Estonia

		Total toxin (extracellular + intracellular) (μg/L)		Intracellular toxin (μg/L)		
		Raw water		Toxin extracted from collected cells		
Sampling location	Date	TR-FRET	IC-TRF	TR-FRET	IC-TRF	LC-MS* [30]
Godby träsk, Finström, Åland Islands, Finland	28.7.2009	0.85	0.68	0.05	0.05	0
Vargata träsk, Lövö Island, Åland Islands, Finland	28.7.2009	57.50	55.25	24.80	21.94	24.5
Nåtö hemviken, Nåtö Island, Åland Islands, Finland	30.7.2009	10.80	9.99	13.02	26.42	8.6
Littoistenjärvi, Kaarina, Finland	26.8.2009	5.41	5.08	16.13	7.48	5.2
Hauninen reservoir, Raisio, Finland	9.6.2009	5.85	7.24	2.48	4.73	11.9
Hauninen reservoir, Raisio, Finland	16.6.2009	10.18	12.08	4.56	11.35	23.6
Hauninen reservoir, Raisio, Finland	4.8.2009	0.52	0.46	0.49	0.29	0.8
Hauninen reservoir, Raisio, Finland	8.9.2009	0.60	0.52	0.78	0.55	1.1
Tuusulanjärvi, Tuusula, Finland	24.6.2009	0.13	0.06	0.02	0.04	0
Maaria reservoir, Turku, Finland	28.7.2009	1.80	1.58	1.71	1.51	1.7
Savojärvi, Pöytyä, Finland	7.8.2009	67.78	55.49	61.32	31.57	40.9
Maaria reservoir, Turku, Finland	11.8.2009	1.44	1.61	0.70	0.64	0.87
Rusutjärvi, Tuusula, Finland	26.8.2009	1.20	1.13	1.43	0.84	0.78
Rusutjärvi, Tuusula, Finland	16.9.2009	2.15	1.49	2.06	1.39	1.1
Lake Peipus, Kauksi beach, Estonia	25.8.2009	0.77	0.82	0.96	0.58	0.52
Lake Peipus, Rannapungerja beach, Estonia	14.8.2009	0.43	0.21	0.31	0.23	0.25
Lake Peipus, Remniku beach, Estonia	14.8.2009	0.69	0.47	0.38	0.26	0.26
Lake Harku, Tallinn, Estonia	6.8.2009	1.20	1.03	1.66	1.27	1.41

LC-MS results were adapted from Savela et al., 2014 [30]

## Discussion

For detection of microcystin or nodularin, compared to the highly expensive high-performance liquid chromatography (HPLC)– or liquid chromatography–mass spectrometry (LC-MS)–based methods, immunoassay-based methods offer advantages in terms of simplicity, cost-effectiveness, and wide accessibility. Furthermore, since raw water can be directly used, immunoassays are especially suitable for handling of large number of samples. Currently, several immunoassays for microcystins and nodularins are commercially available targeting the generic adda residue or specifically targeting the most common microcystin-LR [13, 31]. However, most of the available immunoassays are based on non-competitive methods that require several incubation and washing steps and hence consume several hours to complete the assays. Possibility of reduced assay time from hours to minutes while maintaining the sufficient sensitivity (for example below WHO guideline limit of 1 μg/L of microcystin for drinking water) translates into overall reduced cost as well as rapid decision-making possibilities in a critical situation.

Homogeneous immunoassays, avoiding washing/separation and usually also reagent addition steps, are highly appealing approaches for chemical analytics providing possibilities for the development of simple, rapid, and often cost-effective tools for the detection of specific analytes. In this study, we developed a homogeneous assay for the generic quantitative detection of microcystin and nodularin. This mix-and-measure-type assay combines the advantages of non-competitive immunocomplex-based recognition of the analyte with a sensitive TR-FRET measurement technology. Moreover, due to the unique recognition profile of the immunocomplex forming antibody pair used, the assay allows generic detection of microcystin/nodularin—a large group of related compounds.

The developed TR-FRET assays show maximal S/B ratio of between 10 and 15, depending on the toxin congeners used. These values are significantly higher than those in the two previously reported immunocomplex concept-based TR-FRET assays for morphine [18] and mycotoxin [19], where S/B values between 2 and 4 were observed. One of the factors helping to reach a good performance was the optimization of the labeling conditions for the acceptor-carrying antibody. Best performance was obtained using around 10-fold molar excess of the label. With lower excess, the labels apparently are less likely to hit the sites being at good distance for FRET in the immunocomplex, whereas with higher excess, the self-quenching between the closely located labels probably decreases the FRET efficiency. With the anti-adda Mab, relatively high Eu-labeling degree of 5.5 was obtained by using 1.6 mg/mL Mab and 100x molar excess of Eu-chelate. In theory, maximizing the number of Eu-chelates per antibody would be beneficial for FRET as the fluorescence of Eu-chelates is not prone to self-quenching. However, we decided not to try any further to increase the labeling degree due to the risk of affecting binding properties of the antibody. Indeed, another factor affecting the S/B ratio is the binding affinities of the antibodies involved in the immunocomplex formation. Good functionality of our assay is also reflected as the high sensitivity of detection, which, e.g., for microcystin-LR is ~0.3 μg/L, meeting the WHO guideline limit for microcystins in drinking water.

The assay facilitates rapid measurement; after mixing the reagents with the sample, the signal reaches saturation in 10–15-min incubation at RT. However, already after 2-min incubation, the signal for 1 μg/L of microcystin-LR can be reliably distinguished (Fig. 5). Compared to our previously reported heterogeneous immunoassay for microcystin/nodularin [14], the advantages of the present homogeneous assay include lower sample usage, together with reduced manual work and instrumentation requirements. The assay provides clear advantages for the screening of a large number of samples. However, such a simple and rapid homogeneous assay would, in theory, also very well lend itself for the analysis of individual samples in the field conditions. Concerning this, the main obstacle is the limited availability of portable instruments for the TRF-based measurements.

In comparison to our heterogeneous IC-TRF assay [14], there is more variation in relative signal levels obtained for different toxin congeners in the homogeneous assay. Especially, the signal for nodularin-R is clearly lower than that of microcystins. The results can potentially reflect somewhat lower affinities between the immunocomplex components in the case of nodularin-mediated interaction. Alternatively, the nodularin-mediated interaction might lead to the somewhat different orientation of the interacting antibodies compared to microcystin-based interaction, and the resulting changes in the relative positions for the FRET labels can affect the efficacy of the FRET. In any case, the homogeneous assay could recognize all the tested toxin variants (microcystin-LR, 3-demethylmicrocystin-LR, microcystin-RR, 3-demethylmicrocystin-RR, microcystin-YR, microcystin-LY, microcystin-LF, microcystin -LW, nodularin-R) and most importantly the most toxic and widely reported microcystin-LR variant.

For the practical assessment of the assay, microcystin-LR-spiked water (Table 1) and 18 true environmental water samples (Table 2) were analyzed and the results were compared to those obtained with reference methods (IC-TRF assay and LC-MS). The toxin concentration measured by the TR-FRET assay correlates well with other methods indicating the practical applicability of the assay for the assessment of toxin levels in both direct environmental water and the cell-extracted samples. Overall, water is a very favorable sample matrix for homogeneous FRET-based measurement as it does not, in contrast to, e.g., blood-based sample matrixes, by default contain intensively light-absorbing compounds which could interfere with the excitation, emission, or dipole-dipole coupling–based energy transfer processes in a FRET-based assay. However, should a water sample for some reason have an unusually strong color, a few different dilutions of the samples could, for safety’s sake, be analyzed.

## Conclusions

The presented generic and quantitative homogeneous assay is sensitive enough to be employed in screening of water samples for microcystins below WHO guideline value of drinking water (1 μg/L) and recreational water (24 μg/L). The performance of the assay was demonstrated by analyzing the toxin-spiked sample and real environmental water samples. Being simple and rapid, this mix-and-measure-type assay should be applicable for the analysis of large numbers of water samples for microcystin or nodularin levels. It should also be well-suited for automation and, hereby, useful for high-throughput screening applications.

## Supplementary information

ESM 1 (PDF 335 kb)
