# Supplementary material for: A 15-min non-competitive homogeneous assay for microcystin and nodularin based on time-resolved Förster resonance energy transfer (TR-FRET)
Source: Anal Bioanal Chem. 2021 Jun 3;413(24):6159–70. doi: 10.1007/s00216-021-03375-8 (PMC8440290; doi:10.1007/s00216-021-03375-8)
Supplement: Supplementary file 1 — (PDF 335 kb) [file 216_2021_3375_MOESM1_ESM.pdf]

Analytical and Bioanalytical Chemistry

Electronic Supplementary Material

Article title:

A 15-min non-competitive homogeneous assay for microcystin and nodularin based on time-resolved Förster resonance energy transfer (TR-FRET)

Authors: Sultana Akter and Urpo Lamminmäki (urplammi@utu.fi)

Author affiliation:

Biotechnology, Department of Life Technologies, Faculty of Technology, University of Turku, FI-20520 Turku, Finland.

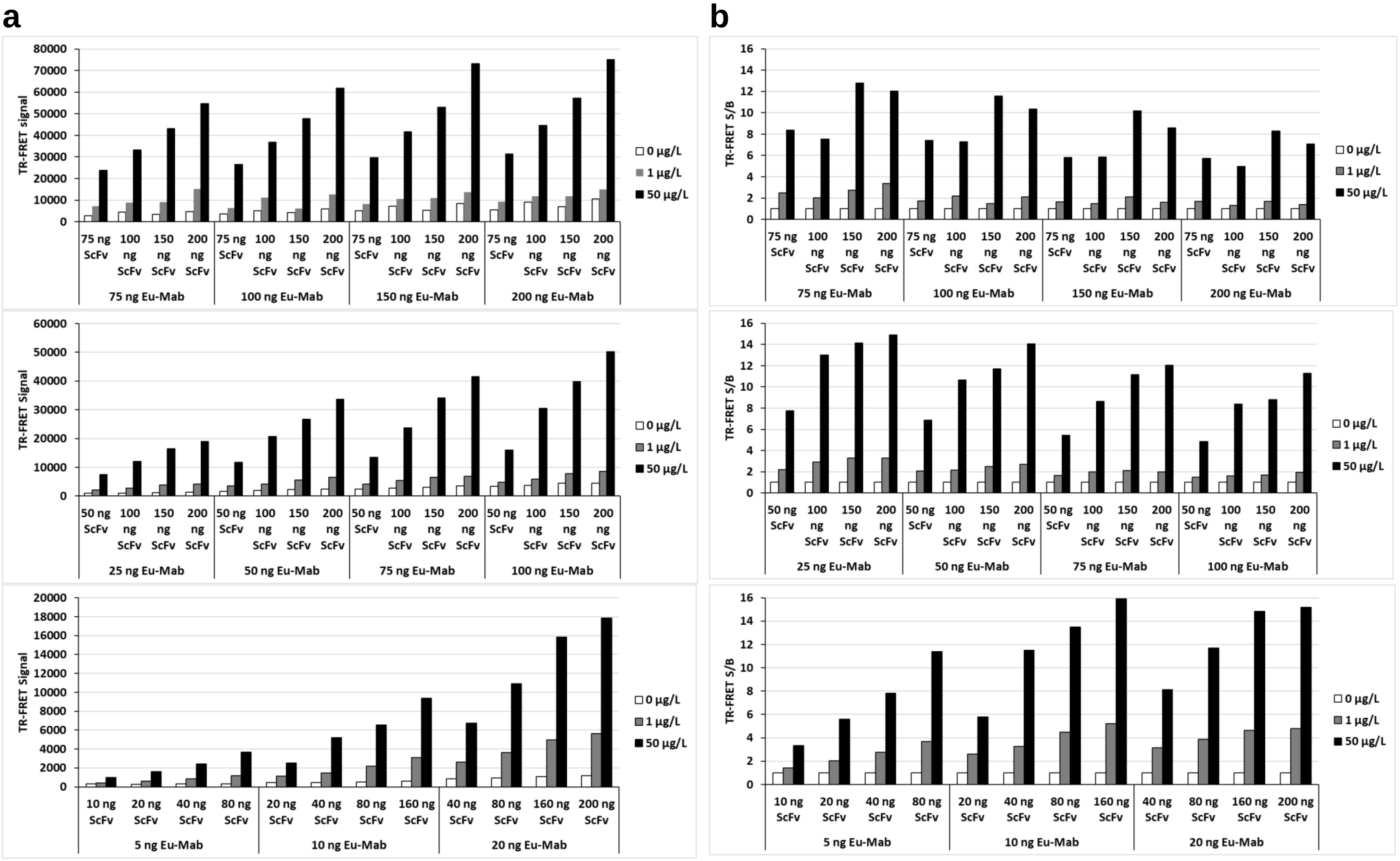
a
b
TR-FRET signal
TR-FRET Signal
TR-FRET S/B
0 µg/L
1 µg/L
50 µg/L
75 ng ScFv
100 ng ScFv
150 ng ScFv
200 ng ScFv
75 ng Eu-Mab
100 ng Eu-Mab
150 ng Eu-Mab
200 ng Eu-Mab
50 ng ScFv
25 ng Eu-Mab
50 ng Eu-Mab
10 ng ScFv
20 ng ScFv
40 ng ScFv
80 ng ScFv
160 ng ScFv
5 ng Eu-Mab
10 ng Eu-Mab
20 ng Eu-Mab

Fig. S1 Optimization of Eu-adda-Mab and AF680-scFv-AP in the homogeneous assay.

Column **a** shows TR-FRET signal (avarage of two replicate measurements) of the sensitized emission of AF680 in the Y axis against the varying amount of Eu-adda-Mab (5-200 ng/well) and AF680-scFv-AP (10-200 ng/well) in presence of 0, 1 or 50 µg/L of microcystin-LR (MC-LR) in 100 µL reaction well (x axis).

Column **b** shows the corresponding TR-FRET signal to blank ratio (S/B) of the sensitized emission of AF680 in the Y axis against the varying amount of Eu-adda-Mab (5-200 ng/well) and AF680-scFv-AP (10-200 ng/well) in presence of 0, 1 or 50 µg/L of microcystin-LR (MC-LR) in 100 µL reaction well (x axis).
